# Generation of a novel disease model mouse for mucopolysaccharidosis type VI via c. 252T>C human *ARSB* mutation knock-in

**DOI:** 10.1016/j.bbrep.2022.101321

**Published:** 2022-08-12

**Authors:** Kosuke Hosoba

**Affiliations:** a Program of Biomedical Science, Graduate School of Integrated Sciences for Life, Hiroshima University, Higashi-Hiroshima, 739-8526, Japan; b Program of Mathematical and Life Science, Graduate School of Integrated Sciences for Life, Hiroshima University, Higashi-Hiroshima, 739-8526, Japan

**Keywords:** Mucopolysaccharidosis type VI, Arylsulfatase B, CRISPR-Cas9 system, Disease model

## Abstract

Mucopolysaccharidosis type VI (MPS VI) is an autosomal recessive lysosomal disorder caused by a mutation in the *ARSB* gene, which encodes arylsulfatase B (ARSB), and is characterized by glycosaminoglycan accumulation. Some pathogenic mutations have been identified in or near the substrate-binding pocket of ARSB, whereas many missense mutations present far from the substrate-binding pocket. Each MPS VI patient shows different severity of clinical symptoms. To understand the relationship between mutation patterns and the severity of MPS VI clinical symptoms, mutations located far from the substrate-binding pocket must be investigated using mutation knock-in mice. Here, I generated a knock-in mouse model of human ARSB Y85H mutation identified in Japanese MPS VI patients using a CRISPR-Cas9-mediated approach. The generated mouse model exhibited phenotypes similar to those of MPS VI patients, including facial features, mucopolysaccharide accumulation, and smaller body size, suggesting that this mouse will be a valuable model for understanding MPS VI pathology.

## Highlights

• We generated a mouse model of the Y85H *ARSB* mutation in humans using CRISPR-Cas9.
• The knock-in mice exhibited phenotypes similar to those of MPS VI patients.
• The mice showed facial features, mucopolysaccharide build-up, and smaller bodies.
• The model could successfully show the correlation between genotype and phenotype.
• These mice could serve as a useful model for novel therapeutic approaches.

## 1 Introduction

Mucopolysaccharidosis type VI (MPS VI), or Maroteaux-Lamy syndrome, is an autosomal recessive genetic and lysosomal disorder resulting from mutation of the *ARSB* gene that encodes lysosomal enzyme N-acetylgalactosamine-4-sulfatase (arylsulfatase B or ARSB, EC 3.1.6.12) [1,2], with a commonly predicted incidence rate of 1 per 100,000 live births [3]. ARSB activity deficiency inhibits the correct degradation of glycosaminoglycan (GAG) dermatan sulfate and chondroitin-4-sulfate. Excessive GAG accumulates in lysosomes and various tissues and induces progressive disorders characterized by coarse facial features, dysostosis, short stature, and cardiopulmonary insufficiency [3,4]. There is no effective curative therapy for MPS VI; hematopoietic stem cell transplantation and enzyme replacement therapy are performed as symptomatic treatments [3].

*ARSB*-knockout animal have been generated to understand the pathogenic mechanisms of MPS VI [1,5]. The observed phenotypes, including low ARSB activity, GAG accumulation in tissues and small body size, were similar to those of patients with MPS VI. These results suggest that ARSB loss-of-function induces MPS VI-like phenotypes in mice. Approximately 12% of patients exhibit nonsense mutations and approximately 18% of patients have deletion or insertion of bases that induce a stop codon in the *ARSB* gene [6]. Previous researches showed that multiple mutations with different severity have been identified in MPS VI patients [3,4,6,7]. Approximately 60% patients have missense mutations [6]. Therefore, investigations using human *ARSB* mutation knock-in mice are needed for a better understanding of MPS VI pathology.

The ARSB protein has two domains: a large N-terminal domain and a small C-terminal domain. This enzyme has two sulfatase domains that are conserved in the sulfatase family [6,[8], [9], [10]]. Previous studies have revealed the 3D structure of human ARSB and its substrate-binding pocket [8,11]. The substrate-binding pocket consists of D53, D54, C91, P93, S94, R95, K145, H147, H242, D300, and K318, suggesting that these amino acids contribute to enzyme activity. Some patients with MPS VI have missense mutations coding in or near the ARSB substrate-binding pocket [6]. However, almost all mutations are located far from the substrate-binding pocket [6,12]. Therefore, the effects of mutations located far from the substrate-binding pocket remain unclear. To understand the pathogenic mechanism of MPS VI, the effects of these mutations must be evaluated. A previous report identified a missense mutation, Y85H (c. 252T > C), in Japanese patients with MPS VI [13]; Y85H was not located in or near the substrate-binding pocket. These patients also showed serious clinical symptoms [13]. Therefore, it is suggested that Y85H knock-in models are more informative than conventional gene knockout animals for understanding the relationship between mutations and phenotypes.

Here, I report knock-in of the Y85H mutation identified using the CRISPR-Cas9 technique. This novel strain was designated as *ARSB*^*em1Hu*^ mouse. The generated mice with biallelic mutations showed facial dysmorphia, shortened body size, and low weight compared to wild-type mice. Biochemical analyses revealed that enzyme activities were decreased in mutant mice, and that GAG accumulated in the tissue of homozygous mutant mice. The designed CRISPR-Cas9 system did not induce off-target mutations in other sulfatases or risk sequences identified in COSMID search. Therefore, the Y85H knock-in mouse is a valuable disease model for understanding MPS VI pathology and furthering therapeutic research.

## 2 Materials and methods

### 2.1 Animals

All animal experiments were performed in accordance with the Hiroshima University guidelines for animal experiments. Before animal experiments, the plans were approved by the Hiroshima University Animal Experiments Committee (Number: C20-45).

### 2.2 Generation of *ARSB*-mutated mice using genome editing technology

F0-mutated mice were generated by Setsuro Tech Co. Ltd. (Japan). C57BL/6J mice were used in this experiment, and electroporation-mediated techniques were performed as described previously [14]. The target sequence for the CRISPR-Cas9 system and single-stranded oligo donor DNA (ssODN) were as follows: *ARSB* (5′-GCTGCACGTAGTAGTTGTCC-3′) and ssODN (5′-cctggatgcgctggcggccggcggcgtggtCCTGGACAACCACTACGTGCAGCcgctgtgcacgccctcgcgg-3′). The Cas9 protein, crRNA, and ssODNs were purchased from Eurofins Genomics K.K. (Japan). The Cas9 protein, crRNA, and tracrRNA were obtained from Integrated DNA Technologies (USA).

The Cas9 protein, (100 ng/μl), tracrRNA (100 ng/μl), and ssODN (200 ng/μl) were introduced into mouse pronuclear fertilized eggs using CUY21EDIT II (BEX). The eggs were cultured and transferred to the oviduct of pseudopregnant female mice. The generated F0 mice were interbred with wild-type mice. Next, F1 mice were interbred and heterozygous and homozygous *ARSB* mutant mice were generated.

### 2.3 Genotyping of generated mice

The generated mice were grown for 4 weeks. After 4 weeks. These crude extracts from ear punch holes were used as PCR templates. The target regions in the genomic DNA were amplified using PCR with specific primer pairs. Primer pairs for other sulfatase genes amplifications were also designed. To evaluate off-target effects, we designed specific primer pairs. The detailed sequences are shown in Supplementary Table.

### 2.4 Micro-CT analysis

CT images of each 10-months-old mouse were acquired using SKY SCAN (BRUKER, USA). Each image was analyzed using NR econ software.

### 2.5 Measurement of ARSB activity

Each tissue of 10-months-old male mice were collected and transferred to a sampling solution (10 mM Tris-HCl pH 7.4, 150 mM NaCl, 5 mM EDTA, and 0.1% Triton X-100) containing a protease inhibitor cocktail (Nacalai Tesque, Japan). Each tissue was sonicated with a Bioruptor II (BMBio, Japan) (30 s × 15). The homogenates were centrifuged at 2,000 g for 3 min at 4 °C. The supernatants were collected. ARSB enzyme activity was measured following the previous study [15]. As a substrate, 4-methylumbelliferyl sulfate was dissolved in assay buffer (0.05 M Na acetate buffer, pH 5.6). To improve the specificity of assay, anti-ARSB antibody treated lysates was prepared. Each extract was incubated with anti-ARSB antibody and Protein A/G-conjugated magnetic beads (Merck Millipore, USA) for overnight at 4 °C. The supernatant was used as sample for assay. Each extract was incubated with 4-methylumbelliferyl sulfate in assay buffer for 30 min at 37 °C. The 125 μM AgNO_3_ was added to assay buffer for inhibition of ARSA activity. Fluorescence was measured at 360 nm (excitation) and 465 nm (emission) using SpectraMax iD3 (MOLECULAR DEVICE, Japan).

#### 2.5.1 Colloid iron staining

Briefly, livers were harvested from each mouse and fixed in 4% paraformaldehyde in PBS overnight. After dehydration, the tissues were embedded in paraffin. Paraffin sections (6 μm) were prepared using a microtome. After deparaffinization and hybridization, colloid iron staining was performed following a previously reported method to detect acid mucopolysaccharides [16]. The sections were treated with 30% acetic acid. The sections were then incubated in colloid iron solution for 60 min at room temperature and washed with 30% acetic acid. After washing, sections were treated with 1% hydrochloric acid and 1% potassium ferrocyanide for 20 min. Collagen was stained with Van Gieson's solution (Scy Tek, USA). Each section was washed with 100% ethanol, embedded in Entellan New (Merck, Darmstadt, Germany), and observed.

### 2.6 Immunofluorescence microscopy

Tissues were harvested from each mouse and fixed in 4% PFA-PBS for overnight. After fixation, each tissue was transferred to 30% sucrose in PBS. The fixed tissues were embedded in O.C.T. compound (Sakura-Finetek, Tokyo, Japan) and frozen. Frozen sections (5 μm) were prepared using a cryostat (Leica Biosystems). After washing with PBS, sections were blocked with 1% BSA-PBS for 30 min at room temperature. The sections were incubated with rabbit polyclonal anti-ARSB (#13227-1-AP, Protein Tech, USA) antibodies in 1% BSA-PBS for 24 h, at 4 °C. Antibody-antigen complexes were detected with Alexa 488-conjugated anti-rabbit secondary antibodies (Invitrogen, USA). After washing, the sections were treated with secondary antibodies and DAPI in 1% BSA-PBS for 60 min at room temperature. After washing with PBS, the sections were embedded in Prolong Diamond anti-fade medium (Invitrogen). For negative controls, primary antibody only and secondary antibody only samples were prepared. Alexa 488 (excitation: 495 nm, emission: 519 nm) and DAPI (excitation: 360 nm, emission: 460 nm) signals were detected by LSM 710 (Carl Zeiss, Germany).

### 2.7 Western blotting

Each protein was analyzed by 10% SDS-PAGE and transferred to PVDF membranes (Merck Millipore, USA) for western blotting. The membranes were blocked in TBST buffer (20 mM Tris pH 7.4, 135 mM NaCl, 2.7 mM KCl and 0.1% Tween-20) with 5% milk. Rabbit anti-ARSB antibody (#13227-1-AP, Protein Tech, USA) and mouse anti-GAPDH antibody (#sc-32233, Santa Cruz, USA) as primary antibodies were diluted by 5% milk-TBST (Dilution was performed following manufacturer's instruction). Each membrane was incubated with primary antibody for overnight at 4 °C. After washing with TBST, membranes were treated with HRP-conjugated secondary antibodies (#NA934 and NA931, GE Healthcare, USA) for 60 min at room temperature (1:20,000 dilution by 2%milk-TBST). After washing, chemiluminescence signals were detected by ChemiDoc™ (BIO-RAD, USA).

## 3 Results and discussion

### 3.1 Generation of Y85H mutation knock-in mouse

**Fig. 1:**
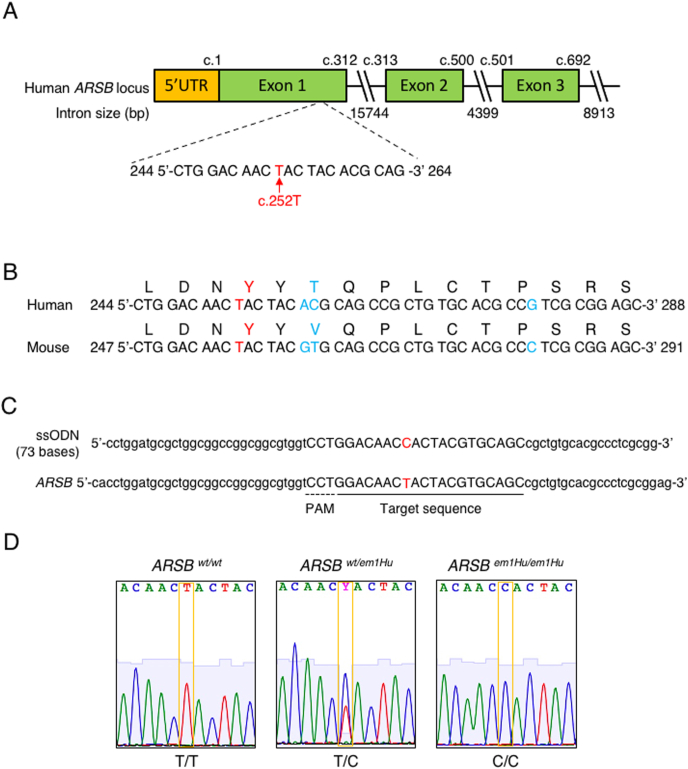
Y85 conservation and strategy of mutation knock-in. (A) Scheme of the human *ARSB* gene; 252T is located in exon 1 of the human *ARSB* gene. (B) Conserved Y85 in mouse. Human and mouse *ARSB* genomic sequences are aligned. The amino acid sequences are also shown on top of the genomic sequences. Y85 is conserved in mice asY86. Red nucleotides and amino acids indicate target bases and amino acids. The blue nucleotides and amino acids indicate non-conserved bases and amino acid. (C) The strategy of CRISPR-Cas9-mediated mutation knock-in. We used 73 ssODN bases as the donor template. The red nucleotides show the targeted bases. The dotted line indicates PAM, and the normal line indicates the target sequence. (D) The sequence results in F2 mice generation. The center panel shows the *ARSB* sequence of heterozygous mutant mice, and the right panel shows the sequence of homozygous mutant mice.

**Supplementary Fig. 1:**
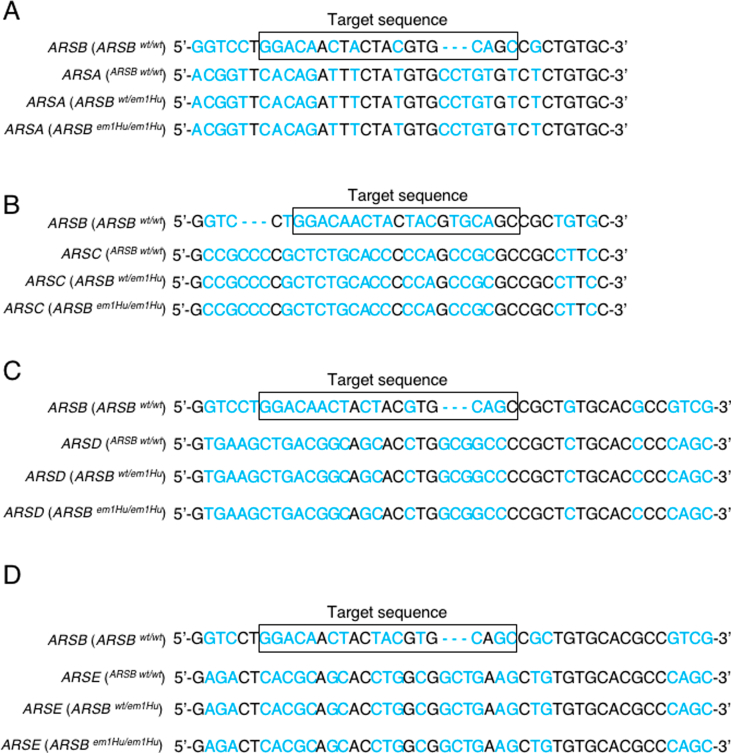
Analysis of *ARSA*, *ARSC*, *ARSD* and *ARSE* loci off-target mutations in the generated mutant mice. (A) Sequence results for the *ARSA* locus. (B) Sequence results of *ARSC* locus. (C) Sequence results for the *ARSD* locus. (D) Sequence results for the *ARSE* locus. Off-target mutations were not identified in these genes. Blue indicates non-conserved bases.

**Supplementary Fig. 4:**
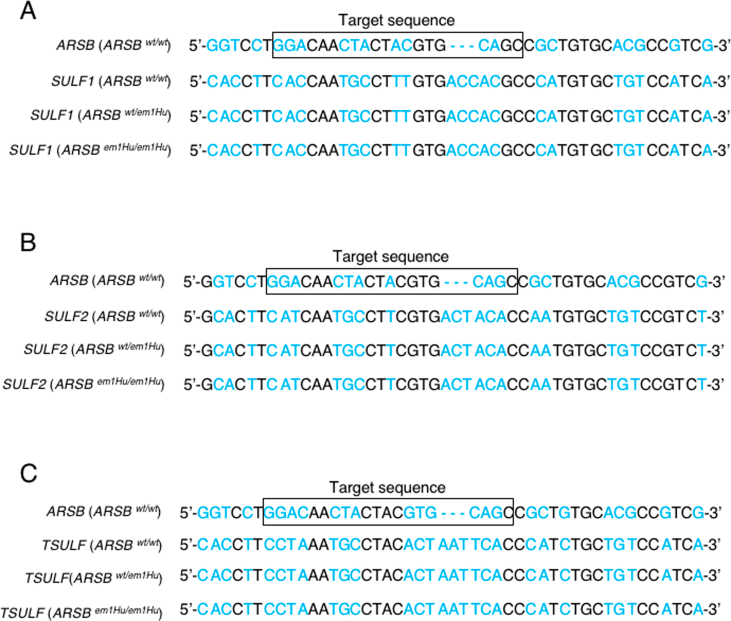
Analysis of *SULF1*, *SULF2* and *TSULF* loci off-target mutations in the generated mutant mice. (A) Sequence results for the *SULF1* locus. (B) Sequence results of *SULF2* locus. (C) Sequence results for the *TSULF* locus. Off-target mutations were not identified in these genes. Blue indicates non-conserved bases.

**Supplementary Fig. 5:**
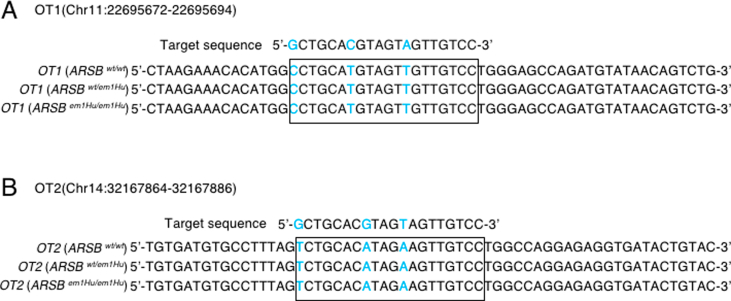
Analysis of off-target mutations in candidate sequences identified via the COSMID search. (A) Sequence results for Chr 11:22695672–22695694 (B) Sequence results for Chr 14:32167864–32167886. Off-target mutations were not detected in the mutant mice. Blue indicates non-conserved bases.

Japanese patients with MPS VI with a p. Y85H (c. 252T > C) missense mutation show severe clinical symptoms. I focused on this mutation as a knock-in candidate. The 252T position is located in exon 1 of the human *ARSB* gene (Fig. 1A). I performed a comparative analysis of the *ARSB* sequence in humans and mice and found that Y85 was conserved in mice as Y86 (Fig. 1B). To generate Y86H knock-in mice, I used CRISPR-Cas9-mediated knock-in. The target sequence and 73 ssODN bases are shown in Fig. 1C. The components of the CRISPR-Cas9 system and ssODNs as donor templates were introduced via electroporation into fertilized eggs. The generated F0 mice were interbred with wild-type mice to confirm germline transmission. Sequence analysis generated heterozygous and homozygous knock-in F2 mice (Fig. 1D). The generated strain was designated as *ARSB*^*em1Hu*^ mouse. As a result of F2 mating, we confirmed the expected 1:2:1 ratio for *wt/wt*, *wt/em1Hu* and *em1Hu/em1Hu*. The two *wt/wt* mice, four *wt/em1Hu* mice and two *em1Hu/em1Hu* mice were generated from mated F2 female mouse (data not shown). The generated mice were maintained as a pedigreed colony. Next, we confirmed the possibility of off-target mutations introduced by CRISPR-Cas9. Sulfatase genes have similar sequences to *ARSB*, and the target region is highly conserved in the sulfatase family. Therefore, we investigated the possibility of off-target mutations of these genes. Possibilities of off-target mutation were not identified by COSMID search. As shown in Supplementary Fig. S1 to Fig. S4, no mutations were detected in other sulfatase gene locus of mutant mice. The COSMID search identified Chr 11:22695672–22695694 and Chr 14:32167864–32167886 as risk sequences for off-target effects of the designed gRNA. These sequences showed an 85% match with the target sequence. Therefore, sequence analysis was performed at these sites. Insertion or deletion of bases was not detected in the generated mice (Supplementary Figs. S5A and B). These results suggest that the risk of off-target mutations was low in our genome editing strategy.

### 3.2 MPS VI-like phenotypes in generated Y86H homozygous mice

**Fig. 2:**
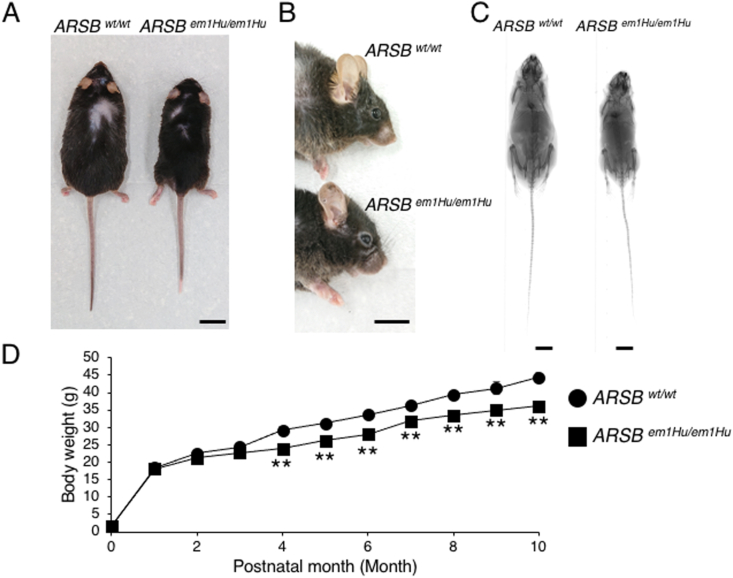
MPS VI-like phenotypes in Y86H homozygous mutant mice. (A) Wild type and homozygous mutant 10-months-old male mice. The body size of mutant mice is smaller than wild type mice. Bar: 2 cm (B) 10-months-old male mutant mice show characteristic facial features. Bar: 2 cm (C) X-ray CT analysis results. Shortened bone size was observed in homozygous mice. Bar: 1 cm (D) The graph of male mice body weight at each time point. The graph shows growth defects in mutant mice. The values represent means ± SD (n＝4). **P < 0.01 (one-way ANOVA, Tukey-Kramer test).

For phenotypic analysis, we observed the morphology of each 10-month-old male mouse. The body size of homozygous mutant mice was smaller than that of wild-type mice (Fig. 2A). Facial features were also confirmed in homozygous mice (Fig. 2B). X-ray CT also showed shortened body size, including bones (Fig. 2C). These experiments were performed under blinded conditions. And the body weight of mice was measured at each time point to record the growth effect of the Y86H mutation. As shown in Fig. 2D, the body weight of homozygous male mice decreased significantly. In this study, four male mice were used in each group. These results suggest that the Y86H mutation induces a severe MPS VI phenotype.

### 3.3 Low ARSB enzyme activity and mucopolysaccharide accumulation in tissues of Y86H mutant mice

**Fig. 3:**
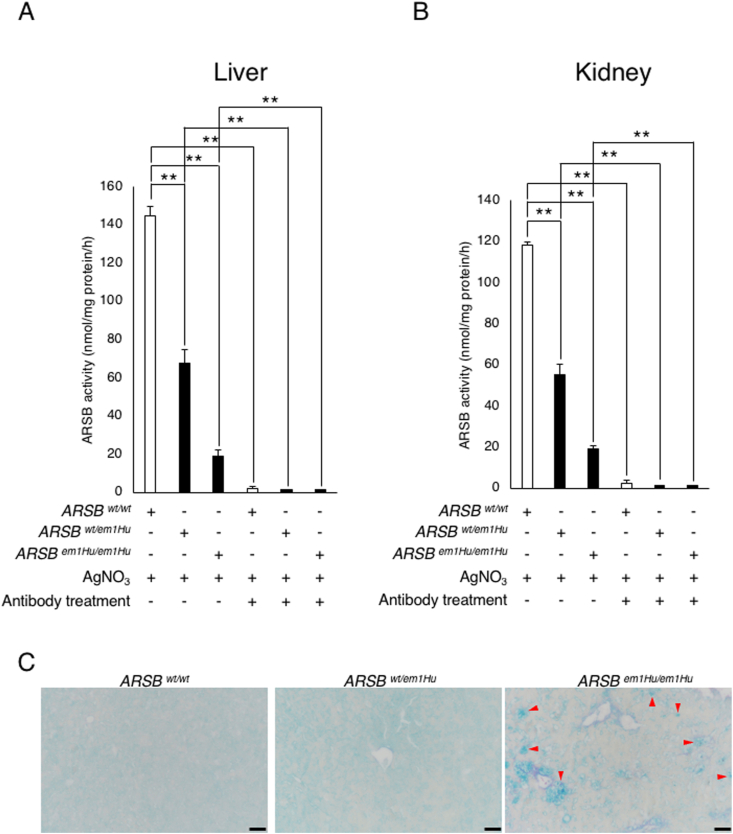
The biochemical and histological analysis of the generated mutant mice. (A) ARSB enzyme activity in liver extracts from each 10-months-old male mouse. Enzyme activity in the liver extract decreased significantly in mutant mice. Activity was not detected in antibody treated samples. The values represent means ± SD (n＝3). **P < 0.01 (one-way ANOVA, Tukey-Kramer test). (B) ARSB enzyme activity in kidney extracts derived from each 10-months-old male mice. Enzyme activity in the kidney extract decreased significantly in mutant mice. Activity was not detected in antibody treated samples. The values represent means ± SD (n＝3). **P < 0.01 (one-way ANOVA, Tukey-Kramer test). (C) Colloid iron staining using liver sections derived from each 10-months-old male mouse. Acidic mucopolysaccharide was detected in the liver tissue sections of homozygous mutant mice. The red arrowheads indicate accumulated mucopolysaccharides. Bar: 50 μm.

It is well known that MPS VI patients show low ARSB enzyme activity. Biochemical experiments were performed to evaluate the ARSB activity in generated mice tissues. Three 10-months-old male mice were used in each group. To inhibit ARSA activity, AgNO_3_ was added to assay buffer. ARSB enzyme activity was decreased in the liver and kidney extracts of heterozygous and homozygous mice (Fig. 3A and B). These results suggest that the 256T > C mutation affects ARSB enzyme activity. 4MU-sulfate was used as substrate of other sulfatases, each extract was treated with anti-ARSB antibody and Protein A/G-conjugated magnetic beads to confirm specificity of assay. As shown in Fig. 3A and B, enzyme activities were not detected in antibody treated sample. Therefore, it was suggested that assay condition was suitable for measurement of ARSB activity.

The accumulation of mucopolysaccharides has also been reported in patients with MPS VI and *ARSB*-deficient animals [5,17]. Next, we observed the accumulation of mucopolysaccharides in the generated mutant mouse using colloid iron staining. The observation was performed under blinded condition. Mucopolysaccharide accumulation was detected in the livers of homozygous mutant mice (Fig. 3C). However, a drastic effect was not observed in heterozygous mutant mice (Fig. 3C), suggesting that monoallelic *ARSB* expression is sufficient to suppress GAG accumulation in lysosomes. Similar clinical features were also observed in patients with severe MPS VI with missense mutations in the *ARSB* gene p. Y85H (c. 252T > C) [13], suggesting that the clinical symptoms of these patients were reproduced in the generated mice. Therefore, it is possible that the mutant mice generated in this study are novel MPS VI models.

### 3.4 Localization and expression of Y86H mutant ARSB protein in liver and kidney tissues

**Supplementary Fig. 6:**
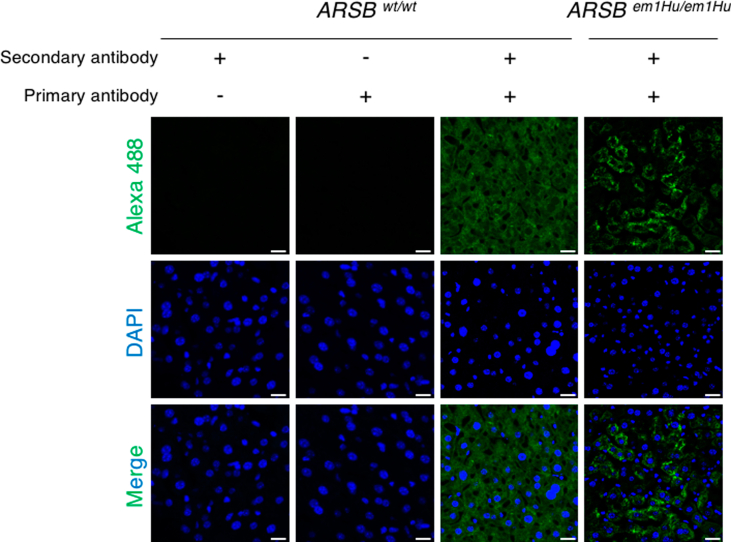
Immunofluorescence microscopy of liver tissues derived from each 10 months-old male mouse using anti-ARSB antibodies. The Y86H mutant protein aggregated in liver tissues. The wild type ARSB protein showed a diffusible localization. Alexa 488 signals were not detected in negative control samples. Bar: 20 μm

**Supplementary Fig. 7:**
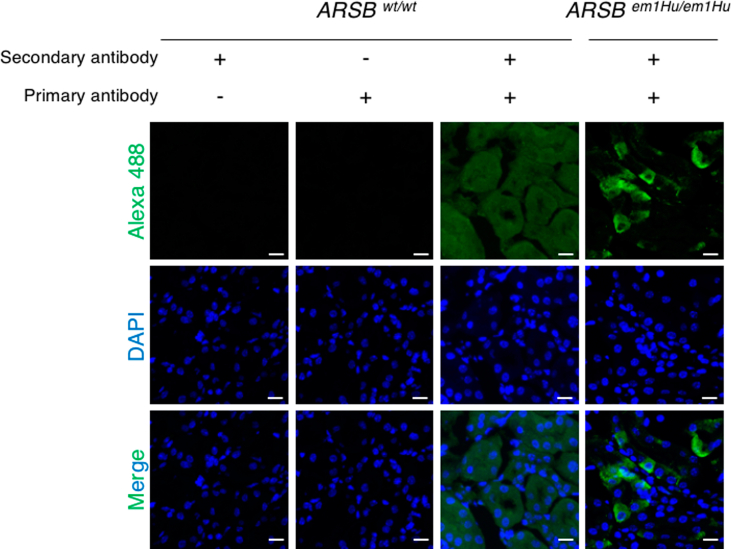
Immunofluorescence microscopy of kidney tissues derived from each 10 months-old male mouse using anti-ARSB antibodies. The Y86H mutant protein aggregated in kidney tissues. The wild type ARSB protein showed a diffusible localization. Alexa 488 signals were not detected in negative control samples. Bar: 20 μm

**Supplementary Fig. 8:**
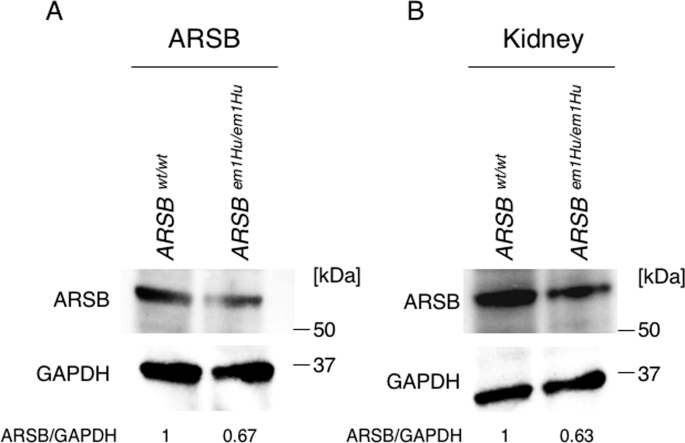
Detection of Y86H mutant protein by western blotting analysis. (A) Y86H mutant protein in liver lysates was detected by western blotting. (B) Y86H mutant protein in kidney lysates was detected by western blotting. Lysates were prepared from each 10-months-old male mice. GAPDH was used for loading control.

**Supplementary Fig. 9:**
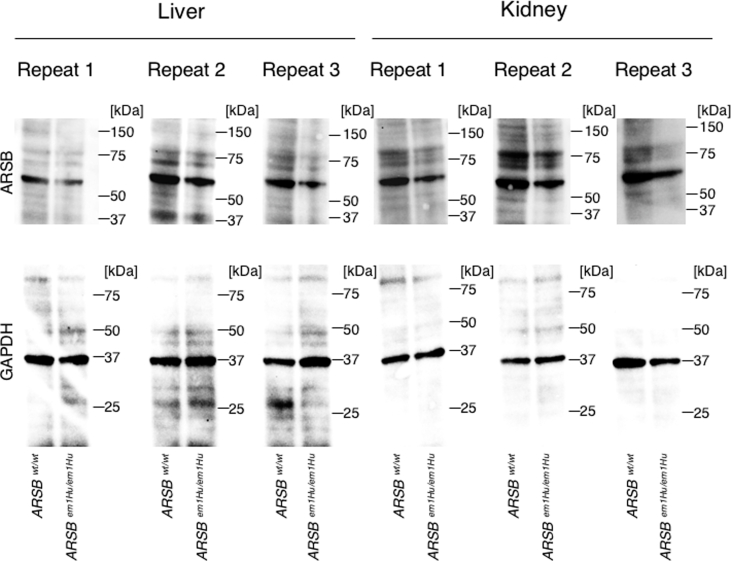
The uncropped images of Supplementary Fig. 8. Three experiments were performed using each tissue lysate from different mice.

Next, we observed the localization of mutant ARSB proteins in Y86H homozygous mice using immunofluorescence microscopy. The diffusible localization of ARSB proteins were shown in wild type mice derived tissues. However, the Y86H mutant protein aggregated in the liver and kidney tissues (Supplementary Figs. S6 and S7). To confirm expression level of Y86H mutant proteins, western blotting was performed. As shown in Supplementary Figs. S8 and S9), signals of ARSB were decreased in homozygous mutant mice derived samples. Previous report showed that mutant ARSB proteins were decreased in patient derived cells [18], it was suggested that Y86H mutant also showed low protein expression comparing wild type. The frequency of diffusible localization was decreased in homozygous mutant mice derived samples (Supplementary Figs. S6 and S7). Therefore, it was suggested that residual mutant Y86H proteins were aggregated in liver and kidney tissues. Y85 is located at the N-terminus of human ARSB and is a component of the large β-strand domain [8,10]. The 3D structure of the ARSB protein was changed by missense mutations [12]. Therefore, it was suggested that structural changes in ARSB were caused by the Y85H (human) and Y86H (mouse) mutations.

### 3.5 Conclusion

To understand the correlation between phenotype severity and mutation site, pleiotropic approaches are desirable. In our study, Y86H mutant mice showed severe phenotypes, suggesting that these mice are novel MPS VI model animals. As a future prospect, comparison of phenotypes using various mutations identified in each domain knock-in mouse will be required to understand the correlation between genotype and phenotype. Hematopoietic stem cell transplantation and enzyme replacement have been performed [5,[19], [20], [21]]. But there are some problems including continuous administration, low penetration in some tissues and physical damages. Recently, it was reported that a β-D-xyloside analog, odiparcil has potential for GAG clearance [22,23]. In these studies, availability of odiparcil was demonstrated *in vitro* and *in vivo* assays including administration test using *ARSB* knock-out mice. The availability of odiparcil as therapeutic drug for MPS VI is examined in phase II trials [24]. The generated MPS VI human mutation knock-in mice are useful models for trials of novel therapeutic approaches.

## Declaration of competing interest

The authors declare the following financial interests/personal relationships which may be considered as potential competing interests.

Kosuke Hosoba reports financial support was provided by 10.13039/501100001691Japan Society for the Promotion of Science.

## Figures and Tables

**Supplementary Fig. 2:**
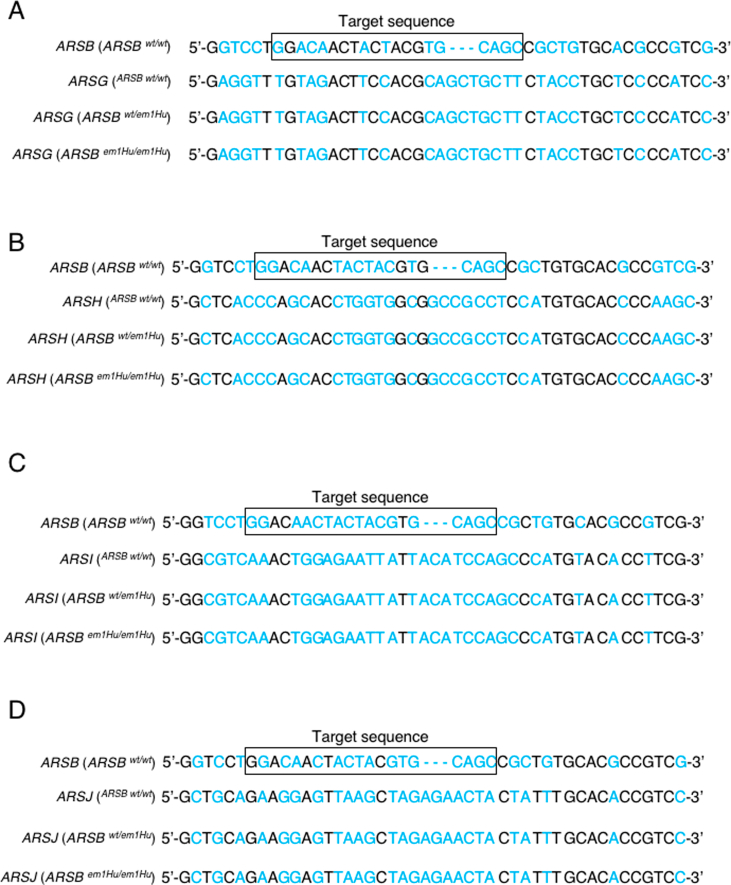
Analysis of *ARSG*, *ARSH*, *ARSI* and *ARSJ* loci off-target mutations in the generated mutant mice. (A) Sequence results for the *ARSG* locus. (B) Sequence results of *ARSH* locus. (C) Sequence results for the *ARSI* locus. (D) Sequence results for the *ARSJ* locus. Off-target mutations were not identified in these genes. Blue indicates non-conserved bases.

**Supplementary Fig. 3:**
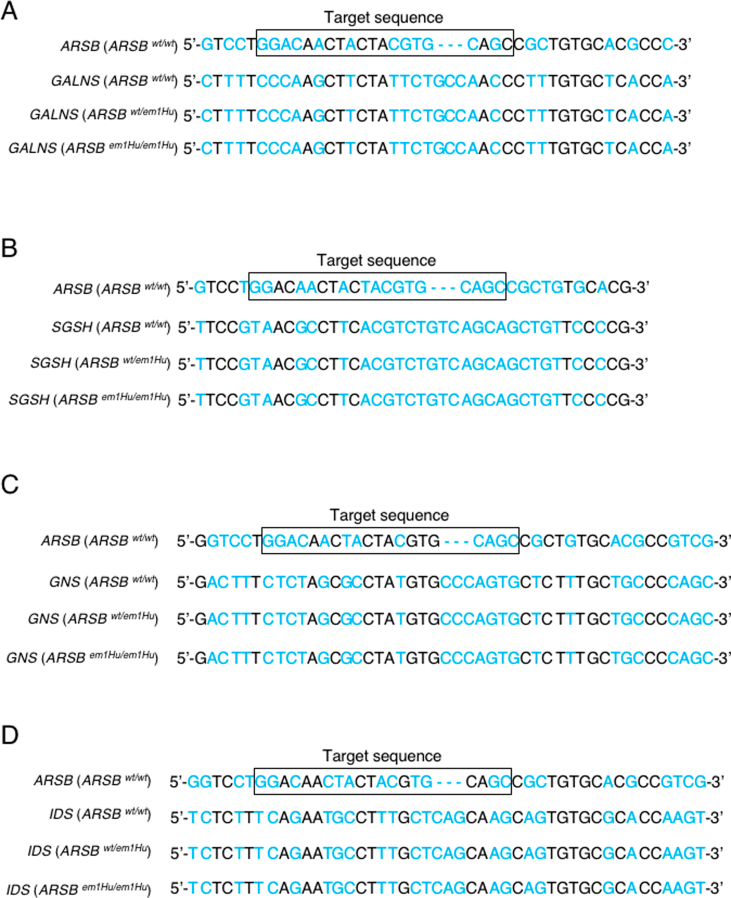
Analysis of *GALNS*, *SGSH*, *GNS* and *IDS* loci off-target mutations in the generated mutant mice. (A) Sequence results for the *GALNS* locus. (B) Sequence results of *SGSH* locus. (C) Sequence results for the *GNS* locus. (D) Sequence results for the *IDS* locus. Off-target mutations were not identified in these genes. Blue indicates non-conserved bases.

## Data Availability

No data was used for the research described in the article.

## References

[1] Valayannopoulos V., Nicely H., Harmatz P., Turbeville S. (2010). Mucopolysaccharidosis VI. Orphanet J. Rare Dis..

[2] Golda A., Jurecka A., Opoka-Winiarska V., Tylki-Szymańska A. (2013). Mucopolysaccharidosis type VI: a cardiologist's guide to diagnosis and treatment. Int. J. Cardiol..

[3] Harmatz P.R., Shediac R. (2017). Mucopolysaccharidosis VI: pathophysiology, diagnosis and treatment. Front. Biosci. Landmark.

[4] Mut M., Cila A., Varlı K., Akalan N. (2005). Multilevel myelopathy in Maroteaux–Lamy syndrome and review of the literature. Clin. Neurol. Neurosurg..

[5] Evers M., Saftig P., Schmidt P., Hafner A., McLoghlin D.B., Schmahl W., Hess B., von F.K., Peters C. (1996). Targeted disruption of the arylsulfatase B gene results in mice resembling the phenotype of mucopolysaccharidosis VI. Proc. Natl. Acad. Sci. U.S.A..

[6] Tomanin R., Karageorgos L., Zanetti A., Al-Sayed M., Bailey M., Miller N., Sakuraba H., Hopwood J.J. (2018). Mucopolysaccharidosis type VI (MPS VI) and molecular analysis: review and classification of published variants in the ARSB gene. Hum. Mutat..

[7] Wraith J.E. (2013). Mucopolysaccharidoses and mucolipidoses. Handb. Clin. Neurol..

[8] Bond C.S., Clements P.R., Ashby S.J., Collyer C.A., Harrop S.J., Hopwood J.J., Guss M. (1997). Structure of a human lysosomal sulfatase. Structure.

[9] Peters C., Schmidt B., Rommerskirch W., Rupp K., Zühlsdorf M., Vingron M., Meyer H.E., Pohlmann R., von Figura K. (1990). Phylogenetic conservation of arylsulfatases. cDNA cloning and expression of human arylsulfatase B. J. Biol. Chem..

[10] Mathew J., Jagadeesh S.M., Bhat M., Udhaya Kumar S., Thiyagarajan S., Srinivasan S. (2015). Mutations in ARSB in MPS VI patients in India. Mol. Genet. Metab. Rep..

[11] Brooks D.A., Robertson D.A., Bindloss C., Litjens T., Anson D.S., Peters C., Morris C.P., Hopwood J.J. (1995). Two site-directed mutations abrogate enzyme activity but have different effects on the conformation and cellular content of the N-acetylgalactosamine 4-sulphatase protein. Biochem. J..

[12] Saito S., Ohno K., Sugawara K., Sakuraba H. (2008). Structural and clinical implications of amino acid substitutions in N-acetylgalactosamine-4-sulfatase: insight into mucopolysaccharidosis type VI. Mol. Genet. Metabol..

[13] Furujo M., Kubo T., Kosuga M., Okuyama T. (2011). Enzyme replacement therapy attenuates disease progression in two Japanese siblings with mucopolysaccharidosis type VI. Mol. Genet. Metabol..

[14] Hashimoto M., Yamashita Y., Takemoto T. (2016). Electroporation of Cas9 protein/sgRNA into early pronuclear zygotes generates non-mosaic mutants in the mouse. Dev. Biol..

[15] Bhattacharyya S., Feferman L., Tobacman J.K. (2016). Restriction of aerobic metabolism by acquired or innate arylsulfatase B deficiency: a New approach to the warburg effect. Sci. Rep..

[16] Mowry R.W. (1958). Improved procedure for the staining of acidic polysaccharides by Muller's colloidal (hydrous) ferric oxide and its combina tion with the Feulgen and the periodic acid Schiff reactions. Lab. Invest..

[17] Yoshida M., Noguchi J., Ikadai H., Takahashi M., Nagase S. (1993). Arylsulfatase B-deficient mucopolysaccharidosis in rats. J. Clin. Invest..

[18] Garrido E., Cormand B., Hopwood J.J., Chabas A., Grinberg D., Vilageliu L. (2008). Maroteaux-Lamy syndrome: functional characterization of pathogenic mutations and polymorphisms in the arylsulfatase B gene. Mol. Genet. Metabol..

[19] Harmatz P., Giugliani R., IV D.S., Guffon N., Teles E.L., Miranda M.C.S., Wraith J.E., Beck M., Arash L., Scarpa M., Ketteridge D., Hopwood J.J., Plecko B., Steiner R., Whitley C.B., Kaplan P., Yu Z.F., Swiedler S.J., Decker C., Group M.V.S. (2008). Long-term follow-up of endurance and safety outcomes during enzyme replacement therapy for mucopolysaccharidosis VI: final results of three clinical studies of recombinant human N-acetylgalactosamine 4-sulfatase. Mol. Genet. Metabol..

[20] Harmatz P., Giugliani R., Schwartz I., Guffon N., Teles E.L., Miranda M.C., Wraith J.E., Beck M., Arash L., Scarpa M., Yu Z.F., Wittes J., Berger K.I., Newman M.S., Lowe A.M., Kakkis E., Swiedler S.J., Group M.V.P.S. (2006). Enzyme replacement therapy for mucopolysaccharidosis VI: a phase 3, randomized, double-blind, placebo-controlled, multinational study of recombinant human N-acetylgalactosamine 4-sulfatase (recombinant human arylsulfatase B or rhASB) and follow-on, open-label extension study. J. Pediatr..

[21] Herskhovitz E., Young E., Rainer J., Hall C., Lidchi V., Chang K., Vellodi A. (1999). Bone marrow transplantation for Maroteaux–Lamy syndrome (MPS VI): long-term follow-up. J. Inherit. Metab. Dis..

[22] Entchev E., Jantzen I., Masson P., Bocart S., Bournique B., Luccarini J.M., Bouchot A., Lacombe O., Junien J.L., Broqua P., Tallandier M. (2020). Odiparcil, a potential glycosaminoglycans clearance therapy in mucopolysaccharidosis VI-Evidence from in vitro and in vivo models. PLoS One.

[23] Entchev E., Antonelli S., Mauro V., Cimbolini N., Jantzen I., Roussey A., Germain J.M., Zhang H., Luccarrini J.M., Lacombe O., Young S.P., Feraille L., Tallandier M. (2022). MPS VI associated ocular phenotypes in an MPS VI murine model and the therapeutic effects of odiparcil treatment. Mol. Genet. Metabol..

[24] Guffon N., Chowdary P., Teles E.L., Hughes D., Hennermann J.B., Huot-Marchand P., Faudot-Vernier E., Lacombe O., Fiquet A., Richard M.P., Abitbol J.L., Tallandier M., Hendriksz C.J. (2022). Oral treatment for mucopolysaccharidosis VI: outcomes of the first phase IIa study with odiparcil. J. Inherit. Metab. Dis..

