# Supplementary material for: Generation of a novel disease model mouse for mucopolysaccharidosis type VI via c. 252T>C human ARSB mutation knock-in
Source: Biochem Biophys Rep. 2022 Aug 12;31:101321. doi: 10.1016/j.bbrep.2022.101321 (PMC9399948; doi:10.1016/j.bbrep.2022.101321)
Supplement: Multimedia component 1 [file mmc1.docx]

Supplementary table

| Primer name | Oligonucleotide sequence (5'→3') |
| --- | --- |
| *ARSB*-forward | CTGCTAAGGATGGGCAAGCT |
| *ARSB*-reverse | GGGCCTCCCTTTTCTTCTCC |
| *ARSA*-forward | CTGGAGCTTCAGAGAGTGGACCTACAGA |
| *ARSA*-reverse | CGAGATGGCGGCACAGAGACACA |
| *ARSC*-forward | GCCGACGACCTGGGCATCGGTGACC |
| *ARSC*-reverse | GAGCCCCGACGACGCCGTGAACAG |
| *ARSD*-forward | AATATCCTACTGATCATGGCGGATGAT |
| *ARSD*-reverse | GGTTTCGTTCTCAGGTAGTCCACC |
| *ARSE*-forward | GACGACCTGGGCATCGGGGATGTCGGC |
| *ARSE*-reverse | TGCAGGAAGGCCCGTGACGTAGCCTTG |
| *ARSG*-forward | GGAGCAAACTGGGCAGAAACAAAGGACAC |
| *ARSG*-reverse | CTCTGCCAAGGTGGTCTCGTTGACTGG |
| *ARSH*-forward | AGCACCCCTAATATTGACCGCCTGGCCAGC |
| *ARSH*-reverse | GTTCACATCGGAGGAGGACGCCAT |
| *ARSI*-forward | GTGGGATACCATGGCTCGGATATCGAGAC |
| *ARSI*-reverse | GGGCTGCCGTGGGCGGATAATGGA |
| *ARSJ*-forward | GCTGATGATCAGGGATTTAGAGATGTG |
| *ARSJ*-reverse | CTTCTGAGGTAGGGTTGCATTGTC |
| *GALNS*-forward | AACTCGTGGAAGCTGCCAGGGTTTGG |
| *GALNS*-reverse | CAGTGTGGCAGTTCTAGGAGACAGAC |
| *SGSH*-forward | GTGTATACAACAACACTGCCATCGCC |
| *SGSH*-reverse | TTGTCAAAAGAGTTGAAGTGATGCAC |
| *GNS*-forward | ACGCCACTGAAGAAAACCAAGGCCCT |
| *GNS*-reverse | GTGTACGGCTCCTGGATCTTCTGCCA |
| *IDS*-forward | CTGGGCTGTTATGGAGACAAGCTAGT |
| *IDS*-reverse | CATACAGGCGGGGGATGGTGGAAAA |
| *SULF1*-forward | GTGGAGCTGGGTTCCCTGCAAGTCATGA |
| *SULF1*-reverse | TGCCACGAGGGAGACGAGCAGTTCTC |
| *SULF2*-forward | ACGGATGACCAGGATGTGGAGCTGGGC |
| *SULF2*-reverse | CCTGCCAGGAGGGCGAGGAACAATT |
| *TSULF*-forward | TTTCAACCAGGAAGTCAGGTAGTAAAACT |
| *TSULF*-reverse | CCAGACCCTTAAAATTATTCCAAGA |
| Chr 11-forward | TCACAGCATTGGGTGACCTAGCTGG |
| Chr 11-reverse | GATGTTTGCAAACTGGTGAAGAGAG |
| Chr 14-forward | AGTTATTTGCCATATAAGTAAACAA |
| Chr 14-reverse | TGTACTGAGGTTGGCCTGAAATGAA |
